# Control Design and Digital Implementation of a Fast 2-Degree-of-Freedom Translational Optical Image Stabilizer for Image Sensors in Mobile Camera Phones

**DOI:** 10.3390/s17102333

**Published:** 2017-10-13

**Authors:** Jeremy H.-S. Wang, Kang-Fu Qiu, Paul C.-P. Chao

**Affiliations:** Institute of Electrical and Control Engineering, National Chaio Tung University, Hsinchu City 300, Taiwan; jeremyw.ece96g@g2.nctu.edu.tw (J.H.-S.W.); gary0713.ece04g@nctu.edu.tw (K.-F.Q.)

**Keywords:** optical image stabilizer (OIS), image blur, nonlinear equations of motion (EOMs), lead-lag controllers, field-programmable gate array (FPGA), mobile camera phones

## Abstract

This study presents design, digital implementation and performance validation of a lead-lag controller for a 2-degree-of-freedom (DOF) translational optical image stabilizer (OIS) installed with a digital image sensor in mobile camera phones. Nowadays, OIS is an important feature of modern commercial mobile camera phones, which aims to mechanically reduce the image blur caused by hand shaking while shooting photos. The OIS developed in this study is able to move the imaging lens by actuating its voice coil motors (VCMs) at the required speed to the position that significantly compensates for imaging blurs by hand shaking. The compensation proposed is made possible by first establishing the exact, nonlinear equations of motion (EOMs) for the OIS, which is followed by designing a simple lead-lag controller based on established nonlinear EOMs for simple digital computation via a field-programmable gate array (FPGA) board in order to achieve fast response. Finally, experimental validation is conducted to show the favorable performance of the designed OIS; i.e., it is able to stabilize the lens holder to the desired position within 0.02 s, which is much less than previously reported times of around 0.1 s. Also, the resulting residual vibration is less than 2.2–2.5 μm, which is commensurate to the very small pixel size found in most of commercial image sensors; thus, significantly minimizing image blur caused by hand shaking.

## 1. Introduction

Image stabilization (IS) technology [1,2,3] has been considered essential for delivering high image quality with minimized image blur in professional cameras. The image blurs are usually caused by the inevitable motion of the camera while shooting. Recently, this IS technology has also been applied to mobile phone cameras to reduce image blur and for meeting new customer demands in the industrial field [1,2,3]. Most of the IS technologies can be categorized as either digital image stabilization (DIS) or optical image stabilization (OIS). DIS technology employs varied techniques of digital image processing to reduce image blurs caused by hand shaking while shooting photos [1,3,4,5,6,7,8]. On the other hand, OIS equips the lens in the camera with mechanical suspension and the associated actuating voice coil motors to move the lens fast enough to compensate for image blurs [2,9,10,11,12,13,14]. As compared to DIS, OIS tries to eliminate image blurs based on basic optics rather than working on imaged digital pixel data as DIS does. When employing DIS, one can significantly lose the original resolution offered by the camera after various digital image processing techniques are applied. Thus, OIS remains viable as an important technology, even with DIS also applied, to tackle the image blur caused by hand shaking as long as the performance of the designed OIS can meet the required specifications [11]. According to their different image stabilization goals, varied OIS structures have been proposed, such as, translational [9,12], rotational [13] as well as a special one using a deformable freeform mirror [14]. The study herein focuses on the translational OIS, since it is most suitable for compensating for the disturbances caused by hand shaking on a mobile phone camera, which is mostly translational.

This study is dedicated to designing, digital implementation and performance validation of the lead-lag controllers for a 2-degree-of-freedom (DOF) translational optical image stabilizer (OIS) installed in mobile camera phones. The related work reported herein includes: (1) the mechanism design of the 2-DOF translational OIS; (2) the establishment of the nonlinear dynamic equations of motion; (3) the design of a simple lead-lag controller based on the established nonlinear equations in order to be implemented via a field-programmable gate array (FPGA) board for fast response; and (4) experimental validation to show the positive performance of the designed OIS and controller to reduce image blur. Compared to all prior reported works, the work presented herein is the first to design a simple lead-lag controller based on complete, complicated nonlinear equations of motion of the OIS, especially including the coupling dynamics between different DOFs being treated as uncertainties during control design. Furthermore, the designed lead-lag controller is easy to implement by using an FPGA chip that is capable of achieving a faster response with the required precision than those reported in prior works [9,12,13,15].

## 2. The OIS Model

### 2.1. OIS Mechanism

**Figure 1:**
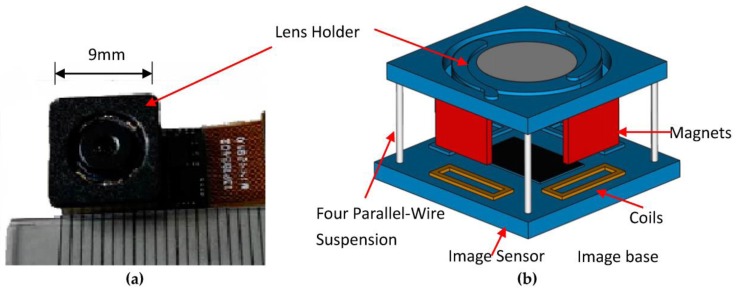
The designed optical image stabilizer (OIS) with 2 translational degrees-of-freedom (DOF), which includes an image base attached with an image sensor, magnets, four-wire suspension and a lens holder. (**a**) a photo; (**b**) a schematic of the OIS structure.

A new OIS is designed for this work with two translational DOFs which has small, slim dimensions of 10.5 mm × 10.5 mm × 4 mm, as shown in Figure 1a. This size is suitable for installation in a common smart phone to hold the focusing lens. The mechanical structure of the OIS mechanism is schematically shown in Figure 1b. The lens is mounted in a lens holder as shown in this figure. Also, the lens holder is suspended to the image base through four parallel vertical wires, with the intention to allow only the movement of the lens relative to the base in two horizontal, translational DOFs for image stabilization against hand shaking. The movement of the lens holder as opposed to the base of the OIS is made possible by the electro-magnetic forces generated between four magnets and coils as shown in Figure 1b. With proper design of the magnet poles and the direction of the current driving through the coils, the generated electro-magnetic forces are able to move the lens in 2 horizontal, translational DOFs to overcome the image blurs due to hand shaking while shooting photos. The resulting important design parameters and expected performance specifications of the designed OIS are listed in Table 1.

To control the movement of the lens holder to the desired position for overcoming negative effects caused by hand shaking, the electromagnetic forces generated by the VCMs are expected to move the lens holder towards the opposite direction of the vibration induced by hand shaking at a required speed. The force generated by the VCMs, ***F***, in a vector can be captured based on basic electromagnetic theory by

(1) $$F=N\cdot i\cdot L\times B_{g},$$

where *N* is the total number of coil loops in a VCM, ***i*** is the vector prescribing the VCM current, *L* is the total length of the coil wires that accounts for generation of electromagnetic forces, and $B_{g}$ is the magnetic flux density that results from design and arrangement of magnets and its yokes.

With the electromagnetic forces in Equation (1) successfully generated by VCMs in a newly-designed OIS structure as shown in Figure 1b, effort was then made to derive the nonlinear equations of motion of the lens and lens holder for the ensuing controller design and experimental validation.

### 2.2. Governing Equations of OIS

**Figure 2:**
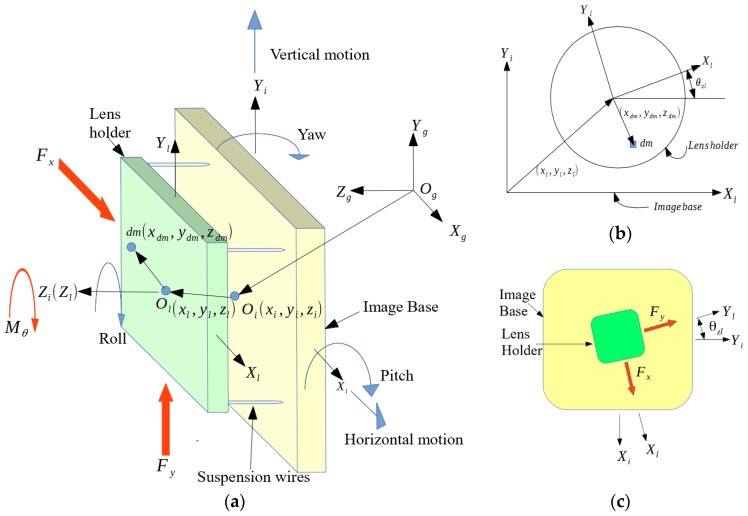
Coordinates and notations that describe the OIS dynamics; (**a**) 3-D view; (**b**) Planar view from the top side view; (**c**) Electromagnetic forces on the centroid of the lens holder.

The equations of motion are derived in this section. This begins with formulating the kinetic and potential energy of the OIS, and then application of Lagrange’s equation to obtain the governing equations of motion for the OIS. For formulating the kinetic and potential energies, two masses are considered. As schematically depicted in Figure 2, they are (1) the image base, to which the active pixel system (APS) of a Complementary Metal-Oxide Semiconductor (CMOS) image sensor is fixed, and (2) the lens and lens holder. Three different coordinates are defined to capture the motion of the lens holder in the OIS relative to ground while shooting photos under hand shaking. The ground reference coordinates are defined as $X_{g}Y_{g}Z_{g}$, the origin of which is $O_{g}$. In addition to $X_{g}Y_{g}Z_{g}$, there are two other coordinate systems, $X_{i}Y_{i}Z_{i}$ and $X_{l}Y_{l}Z_{l}$, which are fixed to the image sensor base and the lens holder, respectively. Note that $X_{i}Y_{i}Z_{i}$ are in fact fixed to the camera case that is vibrating undesirably while shooting photos with hand shaking, causing unintentional shifting of imaged points on the base, eventually leading to image blurs. The VCMs designed and employed in the OIS, as shown in Figure 1, would be expected to move the lens holder, prescribed by the coordinate $X_{l}Y_{l}Z_{l}$, in an opposite direction to compensate significantly for the image blur. Note that the kinematic constraint imposed by the four parallel, vertical wires, as shown in Figure 1b and Figure 2a, restricts the motion of the lens holder to primarily 3 degrees-of-freedom (DOFs), $x_{l}$, $y_{l}$ and ${\theta_{z}}_{l}$, relative to the OIS base.

To design the controller, the governing equations of the OIS need to be derived, which begins by formulating the kinetic and potential energies of mass and inertias in the OIS system based on the coordinates defined in Figure 1. To derive the energies of the image base, in fact, a CMOS image sensor fixed to the camera case, the translational and angular accelerations of $O_{i}$ can be expressed as

(2) $${a_{o}}_{i}=\ddot{x_{i}}\hat{i}+\ddot{y_{i}}\hat{J}+\ddot{z_{i}}\hat{k},\;{{\ddot{\theta }}_{o}}_{i}={{\ddot{\theta }}_{x}}_{i}\hat{i}+{{\ddot{\theta }}_{y}}_{i}\hat{J}+{{\ddot{\theta }}_{z}}_{i}\hat{k},$$

where ${a_{o}}_{i}$ and ${{\ddot{\theta }}_{o}}_{i}$ denote the translational and angular accelerations of the image base, respectively. The Lagrange’s equation [15] applied is

(3) $$\frac{d}{dt}\left(\frac{\partial L}{\partial \dot{r_{i}}}\right)-\frac{\partial L}{r_{i}}=Q_{i},\;i=1,2,\;3,$$

where $r_{i}$ and $Q_{i}$ are generalized coordinates and forces/torques, respectively, and the Lagrangian *L* is

(4) $$L_{o}=T-U=\frac{1}{2}mv_{0}^{2}-U,$$

where $v_{o}$, T and U are the velocity of the lens holder relative to the inertial frame, kinetic and potential energies, respectively. The kinetic energy is due to the motion of the lens holder, while the potential is induced by deflected parallel wires in the OIS, as shown in Figure 2a. Considering the motion of the lens holder is in a moving frame, $X_{i}Y_{i}Z_{i}$, the Lagrangian is no longer Equation (4). Suppose the image base is in a translational velocity V relative to the ground coordinates $X_{g}Y_{g}Z_{g}$, thus $v_{0}=V+v_{1}$, where $v_{1}$ is the velocity of a given mass (dm) of the lens and lens holder relative to the image base in Figure 2a. The Lagrangian in Equation (4) becomes

(5) $$L^{\prime }=\frac{1}{2}mv_{1}^{2}+mv_{1}\cdot V+\frac{1}{2}mV^{2}-U,$$

where

(6) $$v_{1}\cdot V=\frac{d\left(V\cdot r\right)}{dt}-\frac{dV}{dt}\cdot r=\frac{d\left(V\cdot r\right)}{dt}-W\cdot r.$$

In the above Equation (6), r is the displacement of the lens holder relative to the image base. W is the acceleration of the image base in the ground coordinates $X_{g}Y_{g}Z_{g}$. For the non-inertial frame with translational velocity relative to the inertial frame, the Lagrangian is

(7) $$L^{\prime }=\frac{1}{2}mv_{1}^{2}+m\frac{d\left(V\cdot r\right)}{dt}-mW\cdot r+\frac{1}{2}mV^{2}-U,$$

where some terms depending only on time are omitted. In addition, the lens holder possibly rotates slightly with respect to the image base due to asymmetrical mechanism, and $v_{1}=v+\Omega \times r$. Thus, the Lagrangian can be finally formulated as

(8) $$L=T-U=\frac{1}{2}mv^{2}+m\frac{d\left(V\cdot r\right)}{dt}+mv\cdot \Omega \times r+\frac{1}{2}m{\left(\Omega \times r\right)}^{2}-mW\cdot r+\frac{1}{2}mV^{2}-U.$$

where $mv^{2}$ and mW·r are two translational kinetic energies due to translational motion, while mv·Ω×r and $m{\left(\Omega \times r\right)}^{2}$ are rotational kinetic energies. Finally, r, v, Ω, and W of a given differential mass (dm) of the lens holder in Figure 2 can be represented as

(9) $$r=(x_{l}+x_{dm}\cos\;{\theta_{z}}_{l}-y_{dm}\sin\;{\theta_{z}}_{l})\hat{i}+\left(y_{l}+x_{dm}\sin\;{\theta_{z}}_{l}+y_{dm}\cos\;{\theta_{z}}_{l}\right)\hat{J}+\left(z_{l}+z_{dm}\right)\hat{k},$$

(10) $$v=\left({\dot{x}}_{l}-{{\dot{\theta }}_{z}}_{l}x_{dm}\sin\;{\theta_{z}}_{l}-{{\dot{\theta }}_{z}}_{l}y_{dm}\cos\;{\theta_{z}}_{l}\right)\hat{i}+\left({\dot{y}}_{l}+{{\dot{\theta }}_{z}}_{l}x_{dm}\cos\;{\theta_{z}}_{l}-y_{dm}\sin\;{\theta_{z}}_{l}\right)\hat{J}+{\dot{z}}_{l}\hat{k},$$

(11) $$\Omega ={\dot{\theta }}_{x_{i}}\hat{i}+\;{\dot{\theta }}_{y_{i}}\hat{J}+{\dot{\theta }}_{z_{i}}\hat{k},$$

(12) $$W={\ddot{x}}_{i}\hat{i}+{\ddot{y}}_{i}\hat{J}+{\ddot{z}}_{i}\hat{k}.$$

The kinetic energy and potential energies for the lens and lens holder can be derived, respectively, as

(13) $$T=\int \left(\frac{1}{2}v^{2}+\frac{d\left(V\cdot r\right)}{dt}+v\cdot \Omega \times r+\frac{1}{2}{\left(\Omega \times r\right)}^{2}-W\cdot r+\frac{1}{2}V^{2}\right)dm,$$

(14) $$U=\frac{1}{2}k(x_{l}^{2}+y_{l}^{2})+\frac{1}{2}k_{\theta }\theta_{z_{l}}^{2}+gy_{l}m.$$

Equations (9)–(12) are substituted next into Equations (13) and (14) for complete expressions of *T* and U, and this is followed by the application of Lagrange’s Equation (3), yielding the equations of motion along *x*-, *y*- and $\theta_{z}$(roll)-axes as

(15) $$\begin{array}{c} \left({\ddot{x}}_{i}+{\ddot{x}}_{l}-{\dot{\theta }}_{y_{i}}^{2}x_{l}-{\dot{\theta }}_{z_{i}}^{2}x_{l}+{\ddot{\theta }}_{y_{i}}z_{l}-{\ddot{\theta }}_{z_{i}}y_{l}+{\dot{\theta }}_{x_{i}}{\dot{\theta }}_{z_{i}}z_{l}+{\dot{\theta }}_{x_{i}}{\dot{\theta }}_{y_{i}}y_{l}+2{\dot{\theta }}_{y_{i}}{\dot{z}}_{l}-2{\dot{\theta }}_{z_{i}}{\dot{y}}_{l}\right)m\\ +\left({\dot{\theta }}_{x_{i}}{\dot{\theta }}_{z_{i}}+{\ddot{\theta }}_{y_{i}}\right)I_{z}+{\dot{\theta }}_{x_{i}}{\dot{\theta }}_{y_{i}}-{\ddot{\theta }}_{z_{i}}-{\ddot{\theta }}_{z_{l}}\left(I_{x}\sin\;\theta_{z_{l}}+I_{y}\cos\;\theta_{z_{l}}\right)-\\ \left[{\dot{\theta }}_{y_{i}}^{2}+{\left({\dot{\theta }}_{z_{i}}+{\dot{\theta }}_{z_{l}}\right)}^{2}\right]\left(I_{x}\cos\;\theta_{z_{l}}-I_{y}\sin\;\theta_{z_{l}}\right)=F_{x}\cos\;\theta_{z_{l}}-F_{y}\sin\;\theta_{z_{l}}-k_{x}x_{l}-c_{x}\dot{x_{l}}, \end{array}$$

(16) $$\begin{array}{c} \left({\ddot{y}}_{i}+{\ddot{y}}_{l}-{\dot{\theta }}_{x_{i}}^{2}y_{l}-{\dot{\theta }}_{z_{i}}^{2}y_{l}+{\ddot{\theta }}_{z_{i}}x_{l}-{\ddot{\theta }}_{x_{i}}z_{l}+{\dot{\theta }}_{y_{i}}{\dot{\theta }}_{z_{i}}z_{l}+{\dot{\theta }}_{x_{i}}{\dot{\theta }}_{y_{i}}x_{l}+2{\dot{\theta }}_{z_{i}}{\dot{x}}_{l}-2{\dot{\theta }}_{x_{i}}{\dot{z}}_{l}\right)m\\ +\left({\dot{\theta }}_{y_{i}}{\dot{\theta }}_{z_{i}}-{\ddot{\theta }}_{x_{i}}\right)I_{z}-\left[{\dot{\theta }}_{x_{i}}^{2}+{\left({\dot{\theta }}_{z_{i}}+{\dot{\theta }}_{z_{l}}\right)}^{2}\right]\left(I_{x}\sin\;\theta_{z_{l}}+I_{y}\cos\;\theta_{z_{l}}\right)\\ +\left({\ddot{\theta }}_{z_{i}}+{\ddot{\theta }}_{z_{l}}+{\dot{\theta }}_{x_{i}}{\dot{\theta }}_{y_{i}}\right)\left(I_{x}\cos\;\theta_{z_{l}}-I_{y}\sin\;\theta_{z_{l}}\right)=F_{y}\cos\;\theta_{z_{l}}+F_{x}\sin\;\theta_{z_{l}}-k_{y}y_{l}-c_{y}\dot{y_{l}}-mg, \end{array}$$

(17) $$\begin{array}{c} \left(I_{x}\cos\;{\theta_{z}}_{l}-I_{y}\sin\;{\theta_{z}}_{l})({\ddot{y}}_{i}+{\ddot{y}}_{l}-{\dot{\theta }}_{x_{i}}^{2}y_{l}-{\dot{\theta }}_{z_{i}}^{2}y_{l}+{\ddot{\theta }}_{z_{i}}x_{l}-{\ddot{\theta }}_{x_{i}}z_{l}+{\dot{\theta }}_{y_{i}}{\dot{\theta }}_{z_{i}}z_{l}+{\dot{\theta }}_{x_{i}}{\dot{\theta }}_{y_{i}}x_{l}+2{\dot{\theta }}_{z_{i}}{\dot{x}}_{l}-2{\dot{\theta }}_{x_{i}}{\dot{z}}_{l}\right)\\ -\left(I_{x}\sin\;{\theta_{z}}_{l}+I_{y}\cos\;{\theta_{z}}_{l}\right)({\ddot{x}}_{i}+{\ddot{x}}_{l}-{\dot{\theta }}_{y_{i}}^{2}x_{l}-{\dot{\theta }}_{z_{i}}^{2}x_{l}+{\ddot{\theta }}_{y_{i}}z_{l}-{\ddot{\theta }}_{z_{i}}y_{l}+{\dot{\theta }}_{x_{i}}{\dot{\theta }}_{z_{i}}z_{l}+{\dot{\theta }}_{x_{i}}{\dot{\theta }}_{y_{i}}y_{l}+2{\dot{\theta }}_{y_{i}}{\dot{z}}_{l}-2{\dot{\theta }}_{z_{i}}{\dot{y}}_{l})\\ +\left(I_{xz}\cos{\theta_{z}}_{l}-I_{yz}\sin\;{\theta_{z}}_{l}\right)\;\left({\dot{\theta }}_{y_{i}}{\dot{\theta }}_{z_{i}}-{\ddot{\theta }}_{x_{i}}\right)-\left(I_{xz}\sin\;{\theta_{z}}_{l}+I_{yz}\cos\;{\theta_{z}}_{l}\right)\left({\dot{\theta }}_{x_{i}}{\dot{\theta }}_{z_{i}}+{\ddot{\theta }}_{y_{i}}\right)\\ +({\dot{\theta }}_{y_{i}}^{2}-{\dot{\theta }}_{x_{i}}^{2})(I_{xx}\sin{\theta_{z}}_{l}\cos{\theta_{z}}_{l}+I_{xy}(\cos^{2}{\theta_{z}}_{l}-\sin^{2}{\theta_{z}}_{l})-I_{yy}\cos{\theta_{z}}_{l}\sin{\theta_{z}}_{l})\\ +\left({\ddot{\theta }}_{z_{i}}+{\ddot{\theta }}_{z_{l}}\right)I_{\theta }+{\dot{\theta }}_{x_{i}}{\dot{\theta }}_{y_{i}}\left(I_{xx}\cos^{2}\theta_{z_{l}}+I_{yy}\sin^{2}\theta_{z_{l}}-2I_{xy}\sin\theta_{z_{l}}\cos\theta_{z_{l}}\right)-{\dot{\theta }}_{x_{i}}{\dot{\theta }}_{y_{i}}(I_{xx}\sin^{2}{\theta_{z}}_{l}+\\ I_{yy}\cos^{2}{\theta_{z}}_{l}+2I_{xy}\sin\theta_{z_{l}}\cos\theta_{z_{l}})=M_{\theta }-k_{\theta }{\theta_{z}}_{l}-c_{\theta }{{\dot{\theta }}_{z}}_{l}, \end{array}$$

where x, y, and $\theta_{z}$ capture the translational and rotational motions of the lens holder in $X_{i}Y_{i}Z_{i}$. Note that as pointed out previously, the lens holder is constrained motionless relative to the image base along z, $\theta_{x}$ and $\theta_{y}$ by four parallel wires, as shown in Figure 2a. Therefore, the three equations of motion in Equations (15)–(17) suffice to prescribe the dynamics of the lens holder. Also in Equations (15)–(17), $I_{xx}$, $I_{yy}$ and $I_{\theta }$ are the mass moments of inertia of the lens holder, while $I_{xy}$, $I_{xz}$, and $I_{yz}$, are the mass product of inertia of the lens holder about its centroid along the *x*-, *y*- and *z*-axis. $I_{x}=\int_{m}x_{dm}dm$ and $I_{y}=\int_{m}y_{dm}dm$ are the first mass moments of inertia with respect to the *x*- and *y*-axes, respectively. $k_{x}$, $k_{y}$ and $k_{\theta }$ are the equivalent spring stiffness of the four-wire suspension, respectively, along *x*-, *y*- and roll-axes in OIS, while $c_{x}$, $c_{y}$ and $c_{\theta }$ are the equivalent spring damping of the four-wire suspension structure. $F_{x}$, $F_{y}$ and $M_{\theta }$ are the force and moment generated by the VCMs in OIS. Note also that based on the fact of the near symmetry in the OIS structure between *x*- and *y*-directions, $k_{x}$ is assumed equal to $k_{y}$, $c_{x}$ equals $c_{y}$ and $I_{xx}$ equals $I_{yy}$. Moreover, this symmetry also leads to almost zero mass product of inertia and zero first mass moments of inertia of the lens holder over *x*- and *y*-directions. Due to the fact that the translational and rotational displacements of the lens holder relative to the image base are small, the distance between the image base and lens holder, $z_{l}$, is assumed constant and determined by design. Thus, the terms, ${\dot{z}}_{l}$ and ${\ddot{z}}_{l}$, are assumed zeros in Equations (15)–(17), yielding

(18) $$\begin{array}{c} \left({\ddot{x}}_{i}+{\ddot{x}}_{l}\right)m+c_{x}\dot{x_{l}}m+\left[k_{x}-m\left({\dot{\theta }}_{y_{i}}^{2}x_{l}-{\dot{\theta }}_{z_{i}}^{2}\right)\right]x_{l}-2{\dot{\theta }}_{z_{i}}{\dot{y}}_{l}m+\left({\dot{\theta }}_{x_{i}}{\dot{\theta }}_{y_{i}}-{\ddot{\theta }}_{z_{i}}\right)y_{l}m+({\ddot{\theta }}_{y_{i}}+\\ {\dot{\theta }}_{x_{i}}{\dot{\theta }}_{z_{i}})z_{l}\;m=F_{x}\cos\;\theta_{z_{l}}-F_{y}\sin\;\theta_{z_{l}}, \end{array}$$

(19) $$\begin{array}{c} \left({\ddot{y}}_{i}+{\ddot{y}}_{l}\right)m+c_{y}\dot{y_{l}}m-\left({\dot{\theta }}_{x_{i}}^{2}+{\dot{\theta }}_{z_{i}}^{2}\right)y_{l}m+2{\dot{\theta }}_{z_{i}}{\dot{x}}_{l}m+\left({\ddot{\theta }}_{z_{i}}+{\dot{\theta }}_{x_{i}}{\dot{\theta }}_{y_{i}}\right)x_{l}m+({\dot{\theta }}_{y_{i}}{\dot{\theta }}_{z_{i}}-\\ {\ddot{\theta }}_{x_{i}})z_{l}m+k_{y}y_{l}+mg=F_{y}\cos\;\theta_{z_{l}}+F_{x}\sin\;\theta_{z_{l}}, \end{array}$$

(20) $$\left({\ddot{\theta }}_{z_{i}}+{\ddot{\theta }}_{z_{l}}\right)I_{\theta }+c_{\theta }{{\dot{\theta }}_{z}}_{l}+k_{\theta }{\theta_{z}}_{l}=M_{\theta }.$$

Note that a nonzero ${\theta_{z}}_{l}$ does not change the imaging point on the image sensor, and is in fact small due to the nature of the four parallel wires. Thus, the later effort dedicated to controller design can focus on the system with the dynamics prescribed by Equations (18) and (19). In other words, the designed OIS is considered primarily as being of 2 degrees-of-freedom (DOFs), *x* and *y*, to control and then stabilize the image, ignoring the limited motion along an additional DOF of roll, captured herein by ${\theta_{z}}_{l}$. The governing dynamic Equations (18) and (19) can be re-arranged as a system of 2 DOFs for control, in the form of

(21) $$M{\ddot{q}}_{l}+M{\ddot{q}}_{i}+C{\dot{q}}_{l}+Kq_{l}+N+G=TF,$$

where $q_{i}={\left[\begin{array}{cc} x_{i} & y_{i} \end{array}\right]}^{T}$ and $q_{l}={\left[\begin{array}{cc} x_{l} & y_{l} \end{array}\right]}^{T}$ captures the translational motion of the lens holder and the image base, respectively. Note that $q_{i}={\left[\begin{array}{cc} x_{i} & y_{i} \end{array}\right]}^{T}$ in fact captures components associated with *x* and *y* in ***r***, as defined in Figure 2a. *C*, *K* and *M* are the matrices containing the damping coefficient and Coriolis force terms. *K* is the spring stiffness. *M* represents the mass matrices. *N* contains the centrifugal forces. $G={\left[\begin{array}{cc} 0 & mg \end{array}\right]}^{T}$ captures gravitational effect. $F={\left[\begin{array}{cc} F_{x} & F_{y} \end{array}\right]}^{T}$ are electromagnetic forces generated by VCMs for actuation on the lens holder to overcome image blurs caused by hand shaking. $F_{x}$ and $F_{y}$ are in fact generalized forces in ***Q***_i_ in Lagrange’s Equation (3). Note that ***F*** is already prescribed by Equation (1). Expressions of other terms in Equation (21) are

(22) $$M=diag\left[\begin{array}{cc} m & m \end{array}\right],$$

(23) $$C=\left[\begin{array}{cc} c_{x} & -2m{{\dot{\theta }}_{z}}_{i}\\ 2m{{\dot{\theta }}_{z}}_{i} & c_{y} \end{array}\right],$$

(24) $$K=\left[\begin{array}{cc} k_{x}-m\left({\dot{\theta }}_{y_{i}}^{2}+{\dot{\theta }}_{z_{i}}^{2}\right) & m\left({\dot{\theta }}_{x_{i}}{\dot{\theta }}_{y_{i}}-{\ddot{\theta }}_{z_{i}}\right)\\ m\left({\ddot{\theta }}_{z_{i}}+{\dot{\theta }}_{x_{i}}{\dot{\theta }}_{y_{i}}\right) & k_{y}-m\left({\dot{\theta }}_{x_{i}}^{2}+{\dot{\theta }}_{z_{i}}^{2}\right) \end{array}\right],$$

(25) $$N=\left[\begin{array}{c} m\left({\dot{\theta }}_{x_{i}}{\dot{\theta }}_{z_{i}}+{\ddot{\theta }}_{y_{i}}\right)z_{l}\\ m\left({\dot{\theta }}_{y_{i}}{\dot{\theta }}_{z_{i}}-{\ddot{\theta }}_{x_{i}}\right)z_{l} \end{array}\right],$$

(26) $$T=\left[\begin{array}{ccc} \cos\;\theta_{z_{l}} & \sin\;\theta_{z_{l}} & 0\\ -\sin\;\theta_{z_{l}} & \cos\;\theta_{z_{l}} & 0\\ 0 & 0 & 1 \end{array}\right],$$

where *T* is the transform matrix due to small, non-zero ${\theta_{z}}_{l}$ caused by unequal electromagnetic forces of ${\left[\begin{array}{cc} F_{x} & F_{y} \end{array}\right]}^{T}$ on the lens holder. Note that all the terms in Equations (23)–(26) depending on ${\theta_{z}}_{l}$ can be calculated based on Equation (20). With the governing dynamic Equation (21) successfully derived, the controller is designed in the next section based on the derived Equation (21) to achieve image stabilization by moving the lens holder to the desired position while shooting photos with hand shaking.

## 3. Control Design for OIS

### 3.1. Control Objectives

**Figure 3:**
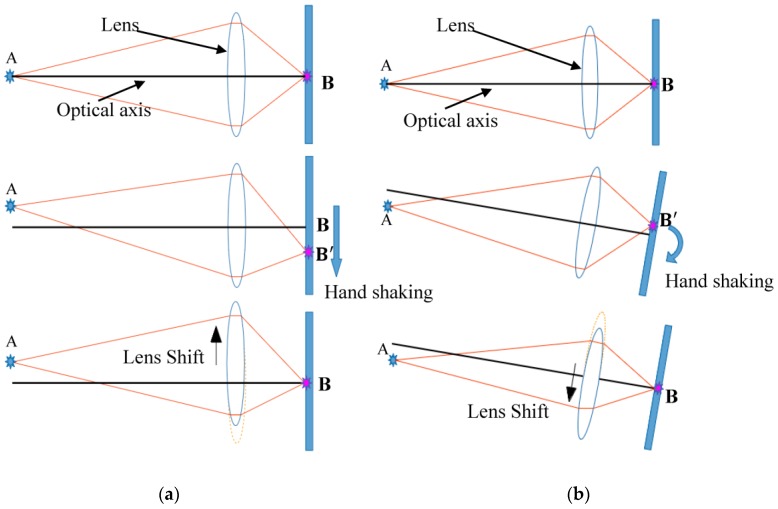
The optical working principles of OIS (**a**) under translational disturbance from hand shaking; (**b**) under rotational disturbance.

The basic optical principle underlying OIS is illustrated in Figure 3, where the movement of the lens and image sensor due to hand shaking are exaggerated for the sake of clarity [13,16]. Note that hand shaking by users while shooting photos on a camera phone induces not only translational but also rotational movements in all six DOFs of the camera. Of all movements, those associated with translational and rotational directions about *x*- and *y*-axes cause many more image blurs than those associated with the *z*-axis. This is due to the fact that the inertial force exerted by photo-shooting is generally not along the *z*-axis, and furthermore, any angular movement about $\theta_{z}$ does not change the imaging point on the sensor plane, leading to no image blur. Therefore, in this study, only the disturbances along *x*- and/or *y*-axes and about $\theta_{x}$- and/or $\theta_{y}$-axes are considered to derive the control objectives of the lens holder.

Figure 3a,b illustrate a lens and its holder in an OIS under disturbance caused by hand shaking while shooting photos along *x*- and/or *y*-axes and/or about $\theta_{x}$- and/or $\theta_{y}$-axes, respectively. In Figure 3a, a downward movement of the camera leads to a downward movement of the image base relative to ground; thus, object A is equivalently moved upward relative to the camera case, including the image base. Thus, the imaged point B is shifted downward by ${\mathrm{BB}}^{\prime }$ to B’, causing imaging blur. To compensate for the blur, the OIS moves the lens upward to re-image point B’ back to origin B, the desired position on the image plane, as shown in the last subfigure in Figure 3a. Similarly, in Figure 3b, point B is also shifted back to its origin, the desired position on the image plane, by the OIS moving the lens downward for some distance, as shown in the last subfigure.

**Figure 4:**
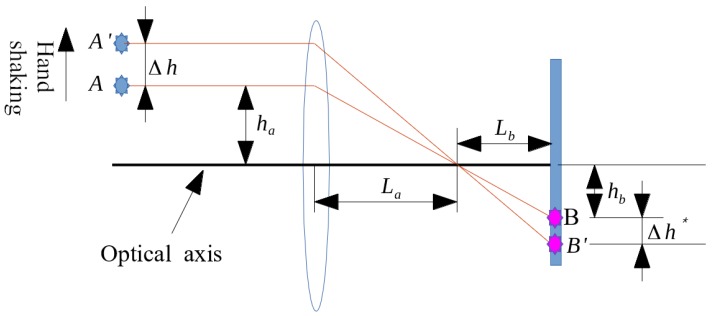
Desired movement of lens holder to compensate for the image blur caused by hand shaking.

With the feasibility of compensating for the imaging blur by moving the lens in the right directions, effort is then made to obtain the desired distance the lens must move to compensate for the image blurs. Figure 4 shows the geometric optics associated with those in Figure 3a, where the camera is under translational disturbance from hand shaking. In the first subfigure of Figure 3a, without hand shaking, object A is imaged on point B on the image plane. While experiencing downward hand shaking, A is shifted from A to A’ by Δh; thus shifting B to B’. To move B’ back upward to its original position, based on geometric optics, the lens needs to be moved upward by $\Delta h^{*}$. This $\Delta h^{*}$ can be actuated by OIS, by satisfying

(27) $$\left(h_{a}+\Delta h-\Delta h^{*}\right)\frac{L_{b}}{L_{a}}-\Delta h^{*}=h_{b}$$

which yields

(28) $$\Delta h^{*}=\Delta h/\left(1+\frac{L_{b}}{L_{a}}\right).$$

Considering that the size of the image plane of the CMOS image sensor is normally much smaller than that of the lens module and the image plane is placed very close to the focal point of the lens module, thus, $L_{a}\gg L_{b}$. Therefore, the above analysis leads to the control objective of the OIS as

(29) $$\Delta h^{*}\approx \Delta h.$$

It is pertinent to note at this point that the control objective in Equation (29) is equivalent to moving the lens in OIS exactly opposite to the movement of the camera due to hand shaking; that is, keeping the lens motionless to ground for image blur compensation. Similar analysis as that outlined above can also be conducted for the case of the camera under rotational disturbance, as shown in Figure 3b. This analysis also arrives at the same control objective in Equation (29) with the assumption of $L_{a}\gg L_{b}$. Note that several optical control objectives similar to Equation (29) were also reported in [17,18]. It has become one of the fundamental, validated rules-of-thumb for OIS in industry. In conclusion, the realistic, combined disturbance along *x*-, *y*-, $\theta_{x}$- and $\theta_{y}$-axes due to hand shaking on the mobile phone camera can be compensated for effectively with the single objective of moving the lens as if motionless to ground by the actuation exerted by OIS.

### 3.2. The Closed-Loop System for the OIS

**Figure 5:**
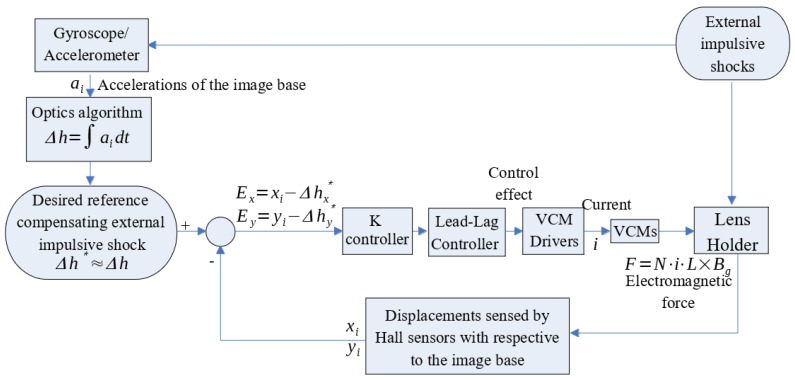
The closed-loop control system of the OIS.

With equations of motion and control objective successfully derived, the closed-loop system for the OIS is ready to synthesize, which is illustrated in Figure 5. In this figure, the four VCMs are driven by a driver circuit which accepts control effort signals from the output of the lead-lag controller. On the other hand, there are Hall Effect sensors installed inside the OIS to sense the relative motion of the lens holder to image base. The signals of the Hall Effect sensors are fed to the controller as feedback to be subtracted from the desired lens movement to compensate for the image blurs by hand shaking. As for the acquisition of the desired lens movement, this is obtained by first sensing the camera movement due to hand shaking by the gyroscope (MPU-6500 by InvenSense, which is supposed to be built into the mobile camera phone). Then the desired lens movement is set up based on Equation (29), i.e., to move the lens in OIS exactly opposite to the movement of the camera due to handshaking; that is, keeping the lens motionless relative to ground at all times. As a result, image blur can be effectively compensated. Note finally, that the VCM driver is implemented by driver circuitry with an H-bridge simplifier inside, which outputs pulse-width modulation (PWM) to regulate the currents through VCMs to be proportional to the output of the lead-lag controller.

### 3.3. System Identification for the OIS

**Figure 6:**
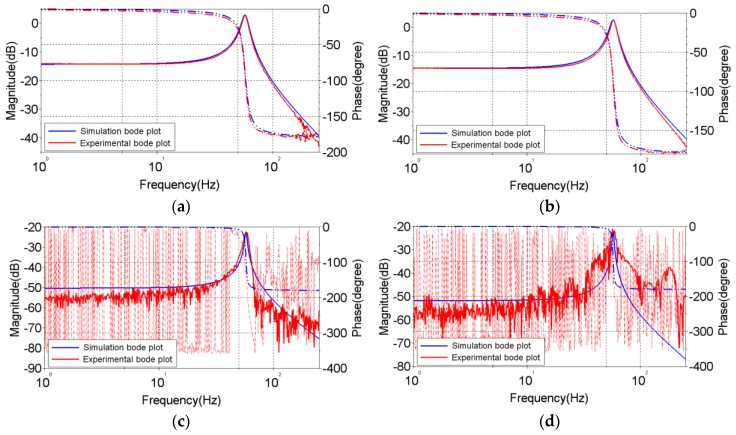
Experimental and simulated Bode plots of the nonlinear Equations (18)–(20) of OIS. (**a**) Experimental and simulated Bode plots of nonlinear OIS in *x* axis with VCM driving along *x* axis, showing a resonance at 57.59 Hz; (**b**) Experimental and simulated Bode plots of nonlinear OIS in *y* axis with VCM driving along *y* axis, showing a resonance at 57.39 Hz; (**c**) Experimental and simulated Bode plots of nonlinear OIS in *x* axis with VCM driving along *y* axis, showing a resonance at 57.60 Hz; (**d**) Experimental and simulated Bode plots of nonlinear OIS in *y* axis with VCM driving along *x* axis, showing a resonance at 57.40 Hz.

The OIS model is identified in this section based on experimental frequency responses of the OIS system; that is, experimental Bode plots. Utilizing a spectrum analyzer, the frequency responses of the designed OIS along different axes can be obtained with swept sinusoidal input signals to drive the circuit of the OIS and using a laser displacement sensor to measure its responses. Measured frequency responses are shown in Figure 6, where four different responses are presented. Figure 6a shows the experimental frequency response in the *x* axis with VCM driving along the *x* axis, exhibiting a resonance at 57.59 Hz. Figure 6b shows experimental the frequency response of the OIS in the *y* axis with VCM driving along the *y* axis, exhibiting a resonance at 57.39 Hz. Figure 6c shows the experimental frequency response of the OIS in the *x* axis with VCM driving along the *y* axis, exhibiting a resonance at 57.60 Hz. Figure 6d shows the experimental frequency response of the OIS in the *y* axis with VCM driving along the *x* axis, exhibiting a resonance at 57.40 Hz. Due to the fact that the resonance in Figure 6a, 57.59 Hz, is very close to Figure 6b, 57.39 Hz, the structure of the OIS, a four-wire suspended structure is highly symmetric between *x* and *y*. On the other hand, the frequency responses in Figure 6c,d represent the coupling dynamics between the *x* and *y* axes, which results from very limited asymmetry in the OIS between the *x* and *y* axes. The amplitudes in Figure 6c,d are significantly smaller than those in the direct dynamics in Figure 6a,b; this indicates that the coupled dynamics of the OIS, corresponding to those cross-diagonal terms in Equations (22)–(26), influence the overall dynamics of OIS much less than direct dynamics, corresponding to the diagonal terms in Equations (22)–(26).

Along with the experimental frequency responses in Figure 6a,b there are theoretical frequency responses simulated by the established nonlinear governing equations of motion (18)–(20) with available information about the varied dimensions of the OIS, the mass of the lens holder, the spring constant estimated by a finite element model, and a simple vibration experiment to obtain the damping coefficient. Table 2 lists the identified parameters of the OIS. It can be seen from Figure 6a,b that the theoretical responses predicted by system Equations (18)–(20) are fairly close to their experimental counterparts, confirming the validity of the established Equations (18)–(20).

### 3.4. Design of a Lead-Lag Controller

A simple lead-lag controller is designed based on the identified model to take advantage of simple digital computation via an FPGA board in order to achieve fast response. As compared to PID, the design process of a lead-lag controller allows one to determine controller parameters for a specific settling time and overshoot, which are very important for OIS control. On the other hand, the fractional controller employed in [15] is, in theory, a better choice than a simple lead-lag controller to achieve the precis, desired closed-loop performance. However, a typical fractional controller would consume much more time than a lead-lag controller to execute its algorithm in a digital chip due to its more complicated nature. For the OIS in a mobile phone camera, a fast settling time is much required.

A typical lead-lag controller is in the form of a lead controller cascaded with a lag controller. On the other hand, since the system of a lens holder and spring wires fixed to the image base is a Type I system, to eliminate steady state error, there must be a free integrator in the lead-lag controller, yielding

(30) $$C\left(s\right)=\frac{k_{p}\left(s-z_{1}\right)\left(s-z_{2}\right)}{s\left(s-p_{1}\right)\left(s-p_{2}\right)},$$

where $z_{1}$ and $p_{1}$ are the zero and pole of the lead controller while $z_{2}$ and $p_{2}$ are the zero and pole of the lag controller, respectively. $k_{p}$ is the proportional gain. The determination of realistic values of $z_{1}$, $p_{1}$, $z_{2}$ and $p_{2}$ starts with setting the final control goals for the closed-loop system of a commercial OIS with a transient period, *t*_s_, less than 0.02 s [18] and the overshoot limited by 5%. The overshoot of 5% leads to the damping ratio, ζ, of 0.6925, and equivalently, a phase margin (PM) of 69.25°. The desired condition of *t*_s_ < 0.02 s together with the determined ζ of 0.6925 decide the poles of the final closed-loop system as in [19],

(31) $$s_{d}=-\varsigma \omega_{n}\pm j\omega_{n}\sqrt{1-\varsigma^{2}}=-200\pm j208.342,$$

for the plant along either *x*- or *y*-directions (both are symmetric), where ω_n_ is determined by the approximation of ω_n_ = 4/(ζ*t*_s_). In the next step, $z_{1}$ is designed as −126.9, close to the pole in Equation (31), to contribute 90° in root loci in the complex plane from open-loop to closed-loop, which is based on the nonlinear governing equations of motion for the system as given in Equations (22)–(26). $p_{1}$ is determined to be −11,600 to achieve more than 180° for robustness to tackle the coupling dynamics as captured by the final closed-loop system. With the steady-state error desired to be under 2%, $k_{p}$ is designed as 3500. In the next step, the lag controller is designed to further minimize the steady-state error without affecting the pre-designed transient period of 0.02 s. To this end, $p_{2}$ is determined as −0.01 to avoid the effects on the pre-designated transient period, while $z_{2}$ is designed as −1 to further minimize steady-state error without affecting the pre-designed transient period of 0.02 s. Finally, we have the lead-lag controller as

(32) $$C\left(s\right)=\frac{3500\left(s+1\right)\left(s+126.9\right)}{s\left(s+0.01\right)\left(s+116,000\right)}.$$

**Figure 7:**
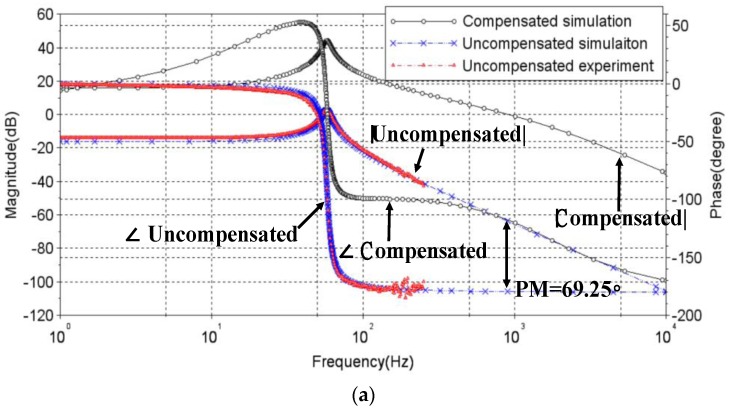

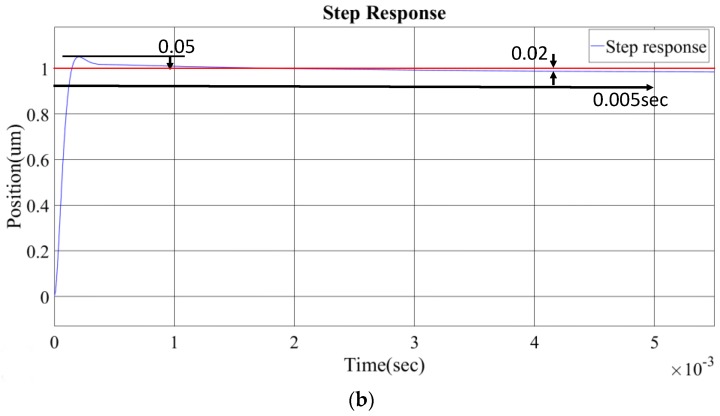
Simulated results with the designed controller applied to the OIS. (**a**) Bode plots for *x*-direction; (**b**) step response along *x*-direction.

Figure 7a shows the Bode plots of experimental/theoretical uncompensated nonlinear plants in *x*-direction and the plant compensated by the designed lead-lag controller in Equation (32). A phase margin of PM~69.25 as pre-designed, can be seen as achieved by the compensated nonlinear plant. Figure 7b shows the simulated step response of the compensated nonlinear plant along *x*-direction, where a transient period much less than 0.005 s and an overshoot around 5% are clearly seen.

## 4. Simulation and Experimental Results

### 4.1. Simulation Results

**Figure 8:**
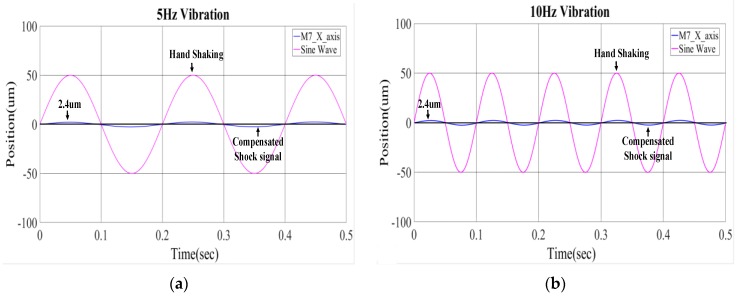

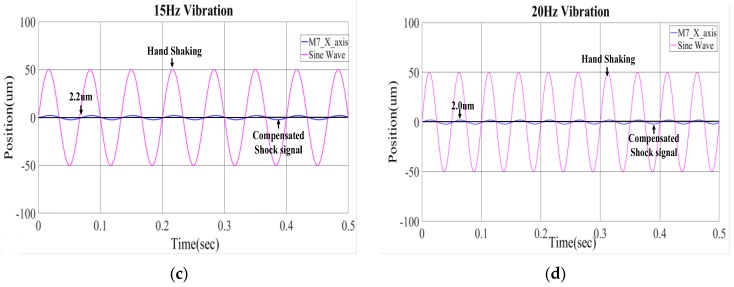
Simulated response of the lens holder motion relative to ground with the designed controller applied to the OIS; (**a**) 5 Hz disturbance in ±50 μm; (**b**) 10 Hz disturbance in ±50 μm; (**c**) 15 Hz disturbance in ±50 μm; (**d**) 20 Hz disturbance in ±50 μm.

Simulations were conducted using MATLAB to verify the performance of the lead-lag controller as designed in Equation (32). In these simulations, disturbances due to hand shaking on the OIS are assumed harmonics at 5, 10, 15, 20 Hz, which in fact correspond to the main harmonics of common hand shakings. Figure 8 shows the simulated response of the lens holder in OIS along *x*-direction relative to ground with the lead-lag controller designed and applied to both the *x*- and *y*-direction of the OIS to suppress the coupling effects from ${\theta_{z}}_{l}$ direction; i.e., those non-zero cross terms in Equations (22)–(26), with some degree of robustness. It is seen from these figures that the designed lead-lag controller is able to control the lens holder at steady state with a residual disturbance within 2.4 μm in amplitude; that is, almost motionless to the ground. This 2.4 μm of residual vibration for the lens holder is actually smaller than the common pixel size of a CMOS/CCD image sensor plane, which is around 2.4–30 μm [20]. Thus, the controller has successfully suppressed the negative effect of hand-shake disturbance on image stabilization, effectively reducing image blurs.

### 4.2. Experimental Results

**Figure 9:**
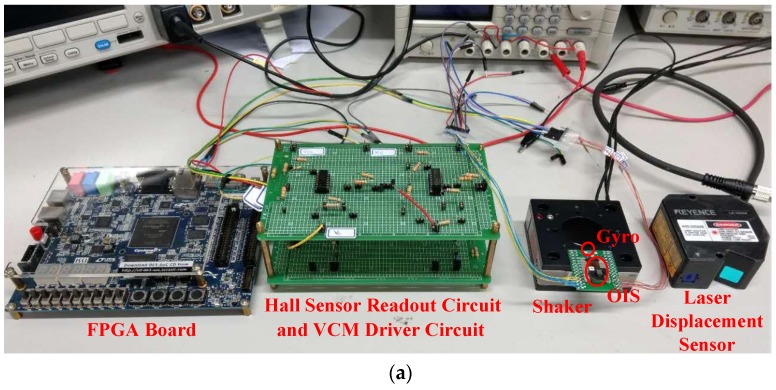

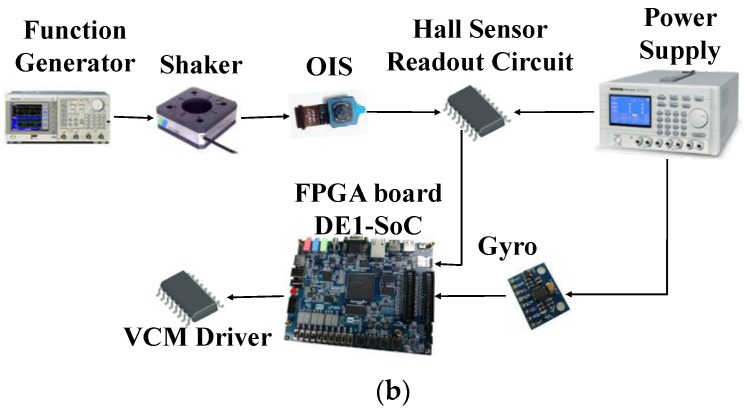
(**a**) Experimental Setup; (**b**) Devices and apparatus in connection.

Experiments were conducted to test the performance of the designed lead-lag controller for stabilizing the lens holder relative to ground, with the aim of reducing imaging blur. Figure 9a shows the setup for conducting the experiments, while Figure 9b illustrates the connection between the device and instruments. In this experimental system, there is an OIS driven by a current drive IC to drive the the lens holder in OIS along *x*- and *y*-directions, a tranlational 2-DOF shaker to simulate hand-shaking disturbance to the OIS, an FPGA board realizing the designed lead-lag controller, and a laser displacement sensor to measure the movement of the lens holder. There are also Hall Effect sensors built into the OIS to measure the real-time motion of the lens holder relative to the OIS image base, and a gyroscope for measuring the motion of the shaker as mimicking hand shake.

**Figure 10:**
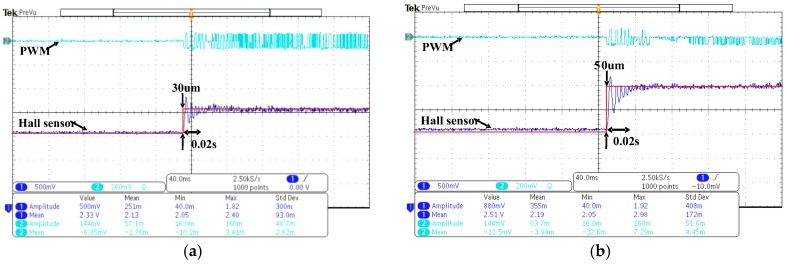
Experimental results of step responses with the designed controller applied to the OIS along *x*-direction; (**a**) Step command of 30 μm; (**b**) Step command of 50 μm.

Figure 10 shows the experimental results of the Hall Effect sensor output for measuring the motion of the lens holder along *x*-direction, with PWM drive signals generated from the current drive IC and step commands of 30 and 50 μm programmed by the current drive IC in Figure 10a,b, respectively. It can be clearly seen from these figures that the lead-lag controller is able to control the lens holder position to the desired 30 and 50 μm within 0.02 s. Thanks to digital implementation of the simple lead-lag controller, this settling time of 0.02 s is much better than prior studies [10] with results of around 0.1 s.

**Figure 11:**
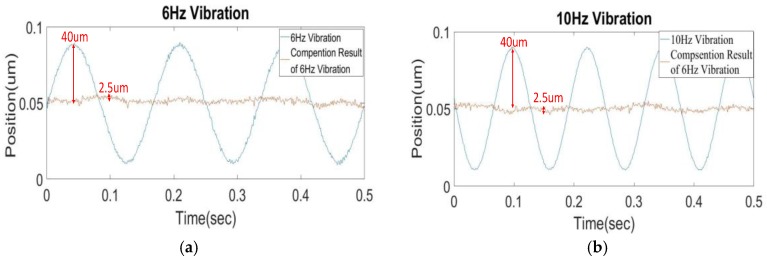

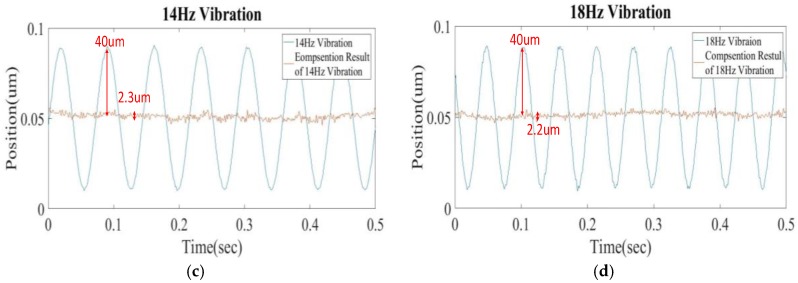
Measured motions of the controlled lens holder with external disturbances programmed by the shaker to mimic major harmonics of a typical hand-shaking disturbance along *x*-direction; (**a**) External disturbance at 6 Hz; (**b**) External disturbance at 10 Hz; (**c**) External disturbance at 14 Hz; (**d**) External disturbance at 18 Hz.

Figure 11 shows the measured movement of the lens holder with external disturbance programmed by the shaker to mimic major frequencies of a typical hand-shaking disturbance along *x*-direction. The disturbance generated by the shaker are at an amplitude of 40 μm at different frequencies of 6, 10, 14, and 18 Hz, corresponding to the main frequencies of typical hand shaking. It can be clearly seen from these figures that as the OIS becomes active, the lens holder is effectively stabilized by the lead-lag controller to be almost motionless to ground within 2.2–2.5 μm, as expected according to the simulations in Figure 8. The residual vibrations are all around 2.2–2.5 μm in magnitude, which is less than or approximately equal to the common pixel size of a CCD/CMOS camera of 2.4–3.0 μm [20]. Thus, it is proven that the designed controller has successfully suppressed the negative effect of hand-shake disturbance on image stabilization in a general mobile phone camera, effectively reducing image blurs.

**Figure 12:**
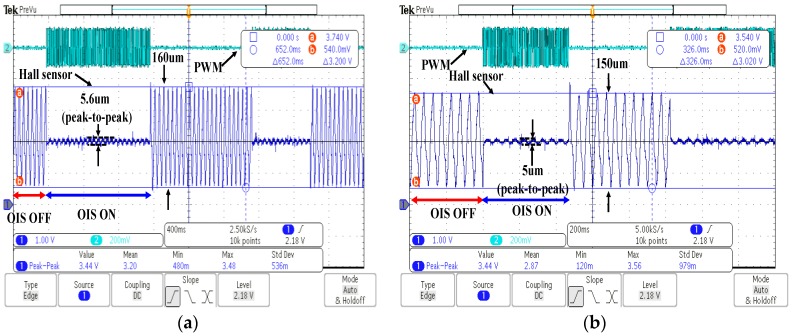
Experimental results with the controller simultaneously applied to *x* and *y* directions of the OIS. (**a**) Experimental result in x direction; (**b**) Experimental result in y direction.

Figure 12a,b show the experimental results with the hand-shaking applied to both *x* and *y* axes of the OIS, while two controllers are also activated on both *x* and *y* axes to stabilize the lens holder. Figure 12a shows the results with the disturbance equally applied by the shaker on the *x* and *y* axes of the OIS, while unequal disturbance is shown in Figure 12b. It is seen from both figures that the controller is able to robustly stabilize the lens holder to be nearly motionless to relative to ground with residual vibration around 5 μm, thus significantly minimizing image blurs.

**Figure 13:**
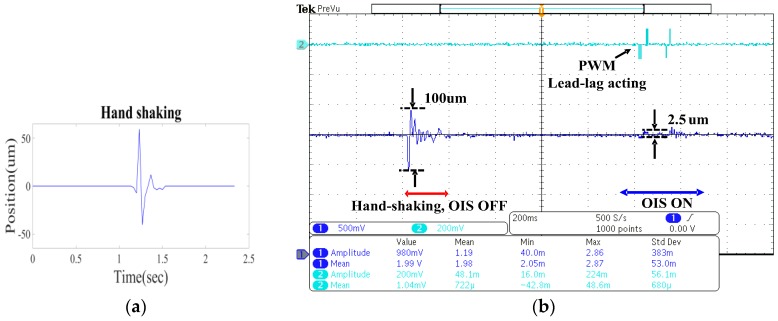
The OIS controller performance under a practical hand shaking; (**a**) The measured hand-shaking as input to the shaker; (**b**) the measured motion of the lens holder in OIS.

Figure 13 shows the experimental results with the hand-shaking programmed by the shaker. Figure 13a shows a measured typical, realistic hand-shaking signal, which in the experiment is fed to the shaker for generating a hand-shaking-like disturbance on the OIS along some axis between *x*- and *y*-directions, while Figure 13b shows the lens holder motion relative to ground measured by the laser displacement sensor. In Figure 13b, the oscillating signal on the left is the lens motion due to realistic hand-shaking with OIS off, while on the right is the motion of the lens holder with OIS on. It can be clearly seen from this figure that the vibration of the lens holder relative to ground is significantly reduced from 100 μm with the OIS initially off, to within 2.5 μm with the OIS on, showing that the OIS is able to stabilize the lens holder relative to ground with very limited image blur.

**Figure 14:**
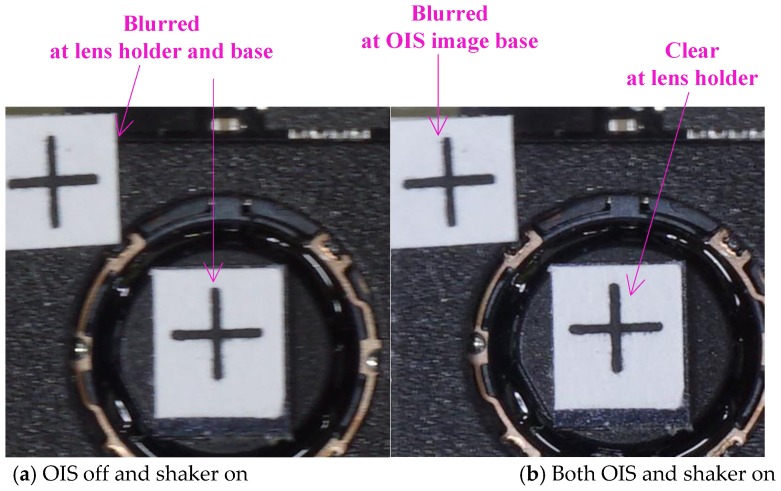
The uncontrolled and controlled photos of the lens holder in an OIS with an exposure time of 0.5 s.

Figure 14 shows experimental images on the OIS image base and its lens holder with the exposure time of another camera set as 0.5 s. Figure 14a shows the situation with the shaker on and OIS off, where both crosses attached to the lens holder and OIS base are blurred due to applied vibration of the shaker at 100 μm. Figure 14b shows the outcome with both the shaker and OIS on; it can be seen from this figure that the designed OIS and lead-lag controller are able to stabilize the lens holder motionless to ground fast enough for a clearer cross image in the lens holder, while the cross attached on the OIS base is blurred as much as in Figure 14a. Based on the fundamental principle proven in Equation (29), and also documented in [16,17], a lens being controlled motionless to ground as shown in Figure 14b is able to perform satisfactory image stabilization against hand shaking.

## 5. Conclusions

A digitally-implemented, simple lead-lag controller was designed and incorporated into a compact OIS. This controller successfully compensates external vibrations induced by hand shaking while shooting photos. This work started with establishing the exact, nonlinear, coupled equations of motion (EOMs) for the OIS, and was followed by designing a simple lead-lag controller based on this nonlinear model, in order to take advantage of simple digital computation via a FPGA board to achieve a fast response. Also, the designed lead-lag controller has robustness and performs satisfactorily to improve blurred images. The experimental results show that the designed controller and OIS are able to stabilize the lens holder to the desired position within 0.02 s in step responses. Thanks to digital implementation of the simple lead-lag controller, this settling time of 0.02 s is much better than previously reported works with a settling time of around 0.1 s. On the other hand, the proposed digital controller is also able to significantly reduce the negative effects of hand shaking on the lens holder suspended in an OIS, resulting in residual vibration less than 2.2–2.5 μm, which is commensurate to the very small pixel size commonly found in most commercial image sensors; thus, significantly minimizing image blurs caused by hand shaking. In the near future, effort will be dedicated to validating the performance of the designed digital lead-lag controller using a CMOS active pixel image sensor (APS) in an OIS.

## Figures and Tables

**Table 1:** Design parameters and specifications of the optical image stabilizer (OIS).

Item	Specifications
Lens holder Diameter	6.7 mm
Lens holder weight	0.5202 g
Rated Load Current	80 mA
Working range	±1 mm
Moving displacement	±1 mm
OIS Dimensions	105 mm × 10.5 mm × 4 mm

**Table 2:** Parameters of optical image stabilizer.

Item	Specifications
Lens holder mass	0.5202 g
Stiffness ($k_{x}$) in *x* axis	67.092 N/m
Stiffness ($k_{y}$) in *y* axis	67.081 N/m
Stiffness ($k_{\theta }$) in roll	$4.189\times 10^{-4}$ Nm
Damping ratios	0.0577
